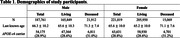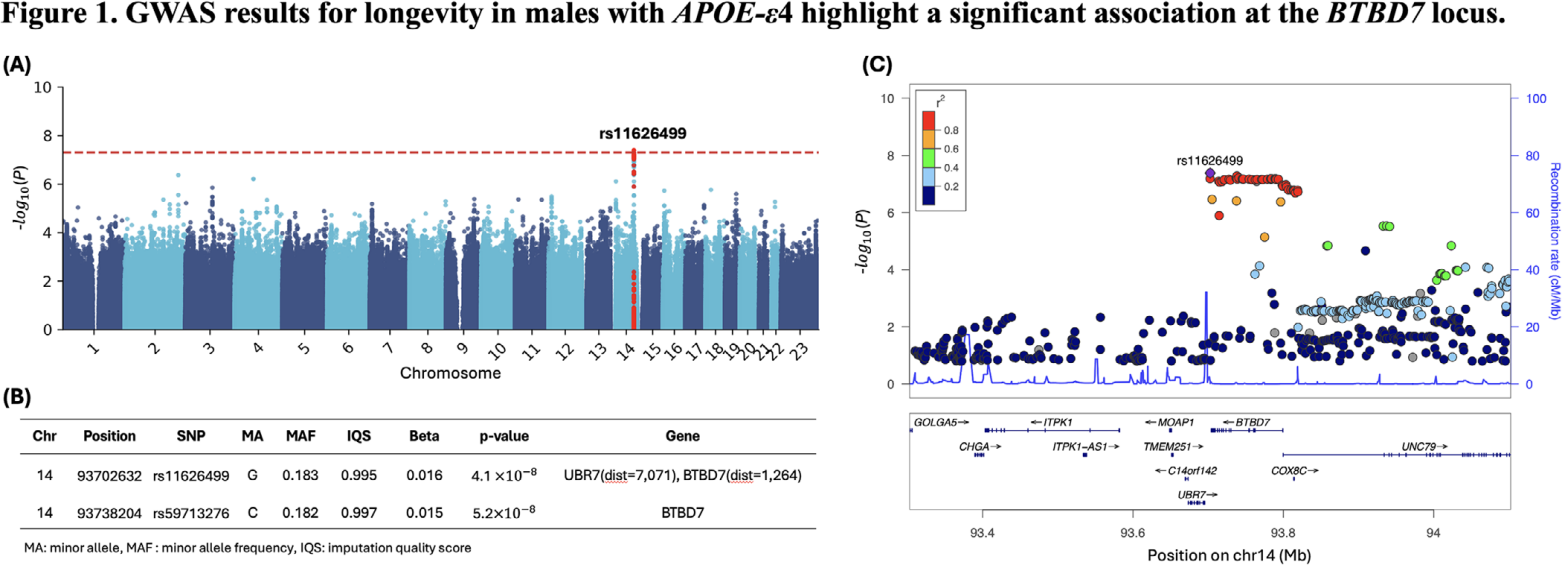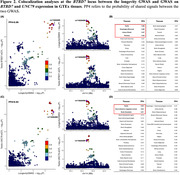# *APOE‐*ε4 interacts with the *BTBD7* testis‐eQTL to reduce longevity in males

**DOI:** 10.1002/alz.095379

**Published:** 2025-01-09

**Authors:** Junyoung Park, Yann Le Guen, Michael D Greicius

**Affiliations:** ^1^ Stanford University, School of Medicine, Stanford, CA USA

## Abstract

**Background:**

The ε4 allele of the *APOE* gene (*APOE*‐ε4) and male sex are well‐established risk factors for reduced longevity. While many genetic studies of longevity have modeled sex and *APOE*, independently, few have considered the interaction of these two risk factors. Here, we model the interaction between *APOE‐ε*4 and sex in a genome‐wide association study (GWAS) of longevity.

**Method:**

This study included a total of 408,780 participants with 36,981 age‐at‐death (male = 21,912, female = 15,069) and 371,799 last‐known‐age (male = 165,849, female = 205,950) from European ancestry participants in UK Biobank (**Table 1**). We performed a GWAS stratified by sex to find significant variant *APOE‐ε*4 genotype interactions associated with longevity using imputed genotype data for common variants (allele frequency>1%). Additionally, we conducted colocalization analyses with expression quantitative loci (eQTL) in GTEx v8 to identify potentially causal genes.

**Result:**

In males (*p* = 4.1×10^−8^), but not in females (*p* = 0.33), we found a significant interaction between *APOE‐ε*4 and rs11626499 near *BTBD7*, which was associated with decreased longevity (**Figure 1**). The survival analysis revealed that in the male *APOE‐ε*4 carrier group, the individuals with the rs11626499_G had a higher risk of mortality (*p* = 2.8×10^−7^, HR = 1.12), but this risk was not observed in the male *APOE‐ε*4 non‐carrier group (*p* = 0.06) or in the female *APOE‐ε*4 carrier group (*p* = 0.14). Furthermore, the GWAS signal colocalizes with associations of *BTBD7* and *UNC79* expression in testis (**Figure 2**).

**Conclusion:**

We identified an interaction between *APOE‐ε*4 and rs11626499 that increases mortality risk only in males. Additionally, rs11626499_G is associated with decreased *BTBD7* and increased *UNC79* expression in testis but not in female‐specific tissues. These findings demonstrate the importance of modeling the interaction between *APOE* and sex in studies of longevity.